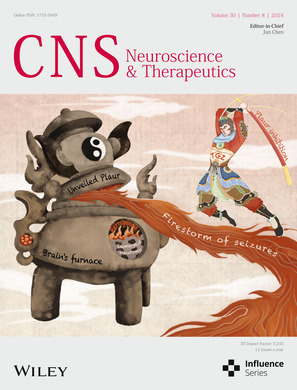# Front Cover

**DOI:** 10.1111/cns.70032

**Published:** 2024-08-29

## Abstract

Cover image: The cover image is based on the article *Unveiling the shield: Troglitazone's impact on epilepsy‐induced nerve injury through ferroptosis inhibition* by Zhi‐Bin Wang et al., https://doi.org/10.1111/cns.14911.